# Genetically Modified Lactic Acid Bacteria in the EU Food Chain: Applications, Benefits, and Risk Assessment

**DOI:** 10.3390/ijms27093759

**Published:** 2026-04-23

**Authors:** Mirco Vacca, Francesco Maria Calabrese, Pasquale Filannino, Maria De Angelis

**Affiliations:** Department of Soil, Plant and Food Sciences (Di.S.S.P.A.), University of Bari Aldo Moro, Via G. Amendola 165A, 70126 Bari, Italy; mirco.vacca@uniba.it (M.V.); francesco.calabrese@uniba.it (F.M.C.); maria.deangelis@uniba.it (M.D.A.)

**Keywords:** lactic acid bacteria, genetically modified microorganisms, genome editing, new breeding techniques, synthetic biology, probiotic engineering, risk assessment, EU regulation

## Abstract

Genetically modified (GM) lactic acid bacteria (LAB) are gaining attention as tools for innovation in the food sector, health applications, and industrial processes. LAB have long been used safely due to their GRAS/QPS status, making them suitable for improving fermentation and synthesizing specific and beneficial metabolites. Advances in genomics and gene editing have significantly expanded the available tools, ranging from classical mutagenesis to site-specific recombination, homologous recombination in non-coding regions, CRISPR-based systems, and food-grade chromosomal integration. These approaches enable the insertion of desired genes and the development of engineered strains with tailored functionalities. GM-LAB are also being studied as live delivery systems for therapeutic molecules, including cytokines, hormones, antimicrobial peptides, and vaccine antigens. Engineered strains of *Lactococcus lactis* and *Lactobacillus* spp. have yielded promising outcomes in applications such as mucosal immunization, modulation of inflammatory and metabolic responses, and inhibition of pathogenic microorganisms, including multidrug-resistant bacteria. From an industrial perspective, several studies highlight their potential for cost-effective recombinant protein production and the synthesis of high-value metabolites through fermentation. However, within the European Union, their use is subject to stringent regulatory oversight, requiring comprehensive molecular and environmental risk assessments, careful evaluation of horizontal gene transfer, and a preference for markerless chromosomal integrations. Despite these constraints, GM-LAB offer significant potential to improve food quality, sustainability, and human health.

## 1. Introduction

**Figure 1:**
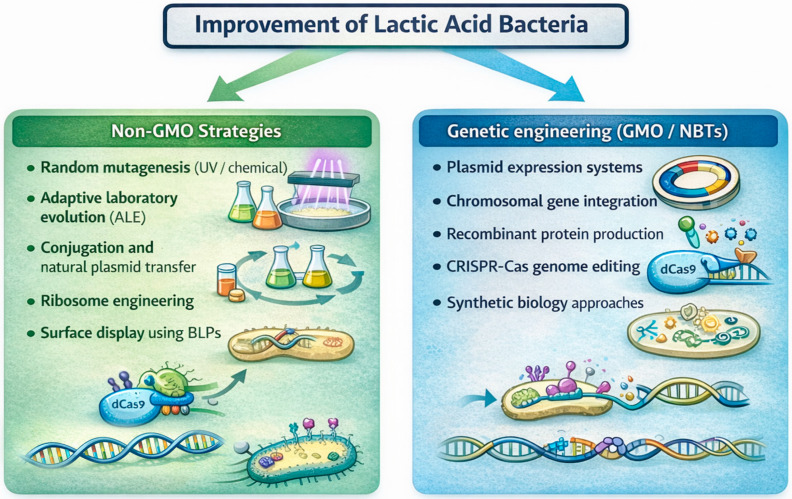
Strategies used for the improvement of lactic acid bacteria (LAB). Classical non-GMO approaches rely on random mutagenesis, adaptive laboratory evolution, or natural gene transfer mechanisms, while modern genetic engineering enables targeted genome modifications through recombinant DNA technologies and genome-editing systems such as CRISPR–Cas.

Global population growth, climate challenges, and an increasing demand for safe, nutritious, and sustainable foods have accelerated the adoption of genetic engineering and new breeding techniques (NBTs) across the agri-food sector [1,2,3]. Together, these approaches have facilitated the development of crops and microorganisms with improved productivity, greater resilience to environmental stresses, and enhanced functional properties [4,5,6,7]. Genetically modified microorganisms (GMMs) play an important role in various applications, including plant growth promotion, contaminated soil bio-remediation, and the design of environmentally sustainable bioprocesses, providing more efficient and ecologically sound alternatives to traditional chemical-based methods [8,9,10,11,12]. In the food sector, GMMs are used either as microbial production platforms for enzymes, vitamins, and novel bioactive compounds or as live cultures purposefully incorporated into food products [13,14,15]. In this framework, lactic acid bacteria (LAB) are key drivers of food fermentation and biopreservation in dairy, meat, cereal, and vegetable products [16,17,18,19,20,21,22], with many species and strains holding a “generally recognized as safe” (GRAS) or Qualified Presumption of Safety (QPS) status [23]. However, advances in genetic engineering have enabled precise modifications of LAB genomes through classical and modern tools (Figure 1), including plasmid-based expression systems, chromosomal integration, CRISPR–Cas systems, and marker-free genome-editing approaches [24,25,26,27]. These innovations have been directed towards the creation of GM-LAB with tailored metabolic capabilities, ranging from enhanced robustness to the production of vitamins or bioactive compounds, as well as their use as platforms for the delivery of therapeutic molecules or mucosal vaccines [28,29,30,31].

Despite these improvements, multiple regulatory challenges persist. The European Union (EU) has implemented a strict framework for GMOs, requiring a detailed risk assessment to ensure a high level of protection for human, animal, and environmental health before any deliberate release or use of GMOs in foods and feed [32]. As a result, although GM-LAB possess significant potential benefits and features that align well with the EU sustainability goals, the EU Green Deal, and the “Farm to Fork” objectives, their use in foods is still limited by regulatory complexity and societal concerns. The widespread adoption of GM-LAB therefore remains constrained by both regulatory and societal factors. Both Directive 2001/18/EC [33] and Regulation (EC) No. 1829/2003 [34] require a case-by-case risk assessment of GMOs, specifically for those used in the food chain. The European Food Safety Authority (EFSA) has issued specific guidance for the risk assessment of GMOs and related products intended for food and feed use, covering molecular characterization, phenotypic properties, toxicology, allergenicity, nutritional impact, and environmental aspects.

Based on these considerations, this review summarizes the main strategies used to improve LAB through classical genetics, modern new breeding techniques (NBTs), and non-GMO approaches, considering their current and potential applications and benefits in the food sector, as well as critically assessing the associated risks and regulatory frameworks, with a focus on the EU context.

## 2. Non-GMO LAB and Their Application in the Food Chain and Human Health

LAB constitute a taxonomic group of Gram-positive, acid-tolerant microorganisms pivotal to food fermentation [35]. Key genera in the food chain include lactobacilli (basonym *Lactobacillus*), *Lactococcus*, *Streptococcus*, *Leuconostoc*, *Pediococcus*, and *Oenococcus*. Their primary metabolic feature relies on the utilization of carbohydrates to synthesize lactic acid (LA), which lowers the pH and its optimum level for the growth of many spoilage microorganisms [23]. In addition to LA, LAB can synthesize different organic acids, exopolysaccharides (EPSs), aroma compounds, bacteriocins, vitamins, and other metabolites contributing to the improvement of food preservation, texture, flavor, and nutritional value [36]. Historically, dairy, meat, and vegetable fermentations harbored natural consortia, adapted to heterogeneous niches, that had been empirically enriched in selected cultures [37,38,39,40]. Therefore, since their introduction as starter cultures during the 20th century, LAB have been increasingly used as processing aids with a safety profile assumed to be suitable for long-term use [41,42,43].

It is also worth considering how genome evolution by means of genome decay, horizontal gene transfer (HGT), and the activity of insertion sequences reveals substantial intra-species variability [44,45,46]. In this regard, evidence of a niche-driven genome evolution emerged from the analysis of the complete genomes of 43 *Lactococcus* strains originating from dairy and non-dairy environments [47]. Dairy isolates showed an extensive genome erosion, including gene loss or pseudogenization of genes involved in carbohydrate metabolism, stress tolerance, and environmental adaptability. In contrast, plant-associated strains maintained more versatile genomes, supporting the hypothesis that dairy strains evolved from plant-adapted ancestors [47]. Similarly, the isolation of LAB from fermented milks obtained from different countries led to the identification of multiple and coexisting lineages of the same LAB species, and it revealed a high intraspecific microdiversity, shaped by mutations and extensive HGT [48]. A detailed inspection of isolate genomes supported the conclusion that ecological interactions within the milk environment have driven the retention or acquisition of clade-specific metabolic traits, with *Lentilactobacillus kefiri* and *Klebsiella ornithinolytica* (basonym *Raoultella ornithinolytica*) acting as the major contributors to the evolutionary pressures shaping LAB genomes [48].

Nonetheless, as noted by Lindgren in 1999, the regulatory status of processing aids was not harmonized within the EU, with some countries applying national provisions and others lacking specific frameworks [49]. This historical context is critical for understanding the regulatory shift that occurred with the emergence of recombinant DNA techniques and their potential application to starter cultures.

### Non-GMO Strain Improvement Approaches

Genomic modification techniques for LAB have steadily improved and progressed from early protoplast-based approaches to efficient electroporation [50]. Genetic transformation crucially depends on the presence of the *ComX* gene, which acts as an alternative sigma factor leading to the expression of genes required for DNA binding, uptake, and recombination in various species of streptococci [51,52]. This indicates that competence can also be induced under specific environmental conditions by enabling *RecA*-dependent homologous recombination and facilitating genome editing without plasmid maintenance [53]. As this condition is compatible with industrial applications [54], such improvements based on non-GMO strains remain widely used—particularly within the EU—in the dairy sector and fermented food industries. Classical methods include random mutagenesis and adaptive laboratory evolution (ALE), which can generate genetic diversity followed by the selection of desirable phenotypes such as those showing enhanced acidification, improved stress tolerance, reduced post-acidification, or increased vitamin production [55,56]. Conjugation enables the transfer of native plasmid advantageous traits, like those involved in lactose metabolism [29,50,57], bacteriophage resistance [58,59,60], or bacteriocin production [61,62,63], without introducing recombinant DNA. Among the approaches that can improve probiotic functionality without foreign DNA integration, ribosome engineering represents one of the most targeted mutagenesis strategies in which antibiotic-resistant mutants are selected, typically via streptomycin or rifampicin, leading to point mutations in ribosomal proteins or RNA polymerase [64,65,66,67].

Non-GMO heterologous surface display systems have been developed as alternatives to recombinant surface displays. In these systems, a recombinant fusion protein containing a peptidoglycan-binding domain (e.g., *LysM*, *SH3b*, *WxL,* or *Cpl-7*) is produced and purified separately and, thereafter, non-covalently anchored to the surface of wild-type LAB or non-living bacteria-like particles (BLPs) obtained by acid or heat treatment of Gram-positive cells [68,69]. Anchor domains recognize and bind specific motifs within the exposed peptidoglycan through non-covalent interactions, allowing for stable surface attachment of the fusion protein. Among these domains, *LysM* is widely used due to it having broad species compatibility and offering a more regulatory-friendly option in specific applications [70,71].

Although non-GMO strain improvement strategies continue to underpin industrial fermentation in the EU because of their regulatory acceptance, their effectiveness is constrained by the stochastic nature of mutagenesis and the limited control over genotype–phenotype relationships. In turn, these limitations have motivated the adoption of classical genetic engineering approaches based on recombinant DNA technologies and, more recently, pushed towards the emergence of precision genome-editing and synthetic biology frameworks.

## 3. Advances in Genetic Engineering of LAB

**Figure 2:**
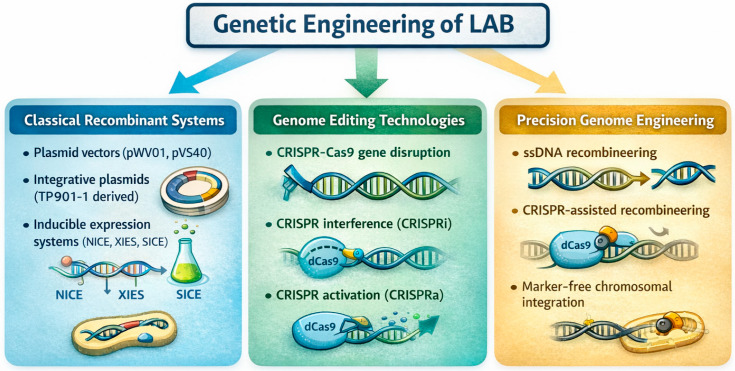
Major genetic engineering tools used in lactic acid bacteria (LAB) modification.

Modern genetic engineering, using diverse toolkits (Figure 2), has rapidly transformed LAB engineering, enabling precise, modular, and efficient genome manipulation [24,72].

Different inducible and secretion-based delivery systems have been developed to optimize the expression of therapeutic proteins, including the Stress-Inducible Controlled Expression module (SICE) [73], Xylose-Inducible Expression System (XIES) [74], and plasmid-based DNA delivery [75]. These approaches improve spatial and temporal control over expression and support targeted mucosal intervention [76]. However, the metabolic burden associated with heterologous gene expression remains a critical limitation, particularly in industrial-scale applications. High-level expression of recombinant proteins can divert essential cellular resources, leading to reduced growth rates, altered metabolic fluxes, and decreased genetic stability [77,78,79]. Consequently, the selection and optimization of expression systems require a careful balance between production efficiency and host fitness, representing a key challenge for the scalability of engineered LAB.

Although Cas9-associated toxicity in several LAB species requires careful tuning of promoter strength and expression systems, modern CRISPR-Cas9 systems allow targeted induction of double-strand breaks, leading to gene disruption or allelic replacement [80,81]. CRISPR interference (CRISPRi) uses catalytically inactive dCas9 and provides a versatile alternative for silencing essential genes without generating lethal DNA breaks [24,82,83]. CRISPR-assisted recombineering integrates phage-derived proteins to enhance homologous recombination, facilitating sequence insertions and other integrative modifications with high efficiency [84,85].

### 3.1. Classical Genetic Engineering of LAB

Chromosomal gene replacements are commonly achieved using temperature-sensitive vectors that promote allelic exchange through double-crossover events, generating stable, marker-free mutants suitable for food chain applications [50,86,87]. These methods enable precise modifications of metabolic pathways and the removal of undesirable traits while maintaining strain genomic stability. Food-grade plasmid systems introduced in the early 2000s laid the groundwork for the safe genetic modification of LAB intended for food applications [28,50,88,89,90,91]. Plasmids such as pWV01, pVS40, and pCI305 made it possible to perform cloning using exclusively LAB-derived genetic elements, thereby avoiding the use of antibiotic-resistance markers [28,50,89,92]. In addition, food-compatible selection strategies were implemented through complementation markers based on carbohydrate utilization (e.g., sucrose or xylose operons) or amino acid auxotrophy, such as D-alanine dependence generated by *alr* deletion [93,94,95,96,97]. Integrative plasmids, such as those derived from *TP901-1*, allow marker-free chromosomal integration through double-crossover events, significantly increasing genetic stability [72,98,99].

### 3.2. Genome-Editing and Synthetic Biology Tools

Single-stranded DNA (ssDNA) recombineering is an efficient and flexible LAB strategy, enabling RecT-dependent recombination through the delivery of short synthetic oligonucleotides at the replicative fork. This approach permits precise genome modification without the need for plasmid cloning or antibiotic selection markers [100,101]. However, several technical limitations remain. Editing efficiency may vary significantly among LAB species due to differences in recombinase compatibility, DNA uptake mechanisms, and host mismatch repair (MMR) systems, which often recognize and correct introduced mutations. Furthermore, recombineering efficiency depends on the timing of oligonucleotide incorporation during lagging-strand DNA replication and may require transient inhibition of the host MMR pathway to increase editing rates. Despite these challenges, when combined with CRISPR-based counterselection, this method can yield highly enriched mutant populations, enabling multiplex genome editing and significantly reducing the time required for strain optimization [64]. This methodology has been shown to be effective in *Lactococcus (L.) lactis* and *Limosilactobacillus (Ls.) reuteri*, allowing the generation of scarless and marker-free mutations suitable for both industrial and clinical applications [64,102,103,104]. Non-edited cells are selectively eliminated through CRISPR–Cas9 targeting, resulting in a strong enrichment of modified variants [105].

CRISPR–Cas systems are widely distributed among LAB and are particularly represented by types I and type II CRISPR–Cas systems, which originally evolved as defense mechanisms against bacteriophages and mobile genetic elements [106,107,108]. However, at present, these have become fundamental tools for genetic engineering in the food and probiotic sectors, enabling the development of editing strategies that do not require the stable incorporation of exogenous DNA. CRISPR–Cas systems are based on arrays consisting of short repeat sequences interspersed with variable spacers derived from foreign genetic elements. These CRISPR arrays are transcribed into polycistronic precursor RNAs, which are subsequently processed into individual CRISPR RNAs (crRNAs), each guiding the Cas nuclease to a specific target sequence. In genome engineering applications, synthetic CRISPR constructs mimic this natural architecture by combining repeat–spacer units designed to recognize specific genomic loci. The polycistronic nature of these transcripts allows simultaneous targeting of multiple genes, enabling multiplex genome editing and coordinated modification of diverse metabolic pathways.

In addition to genome editing, CRISPR-based tools have also been extended to modulate gene expression through CRISPR interference (CRISPRi) and CRISPR activation (CRISPRa), further expanding the range of strategies available for transcriptional regulation. The adoption of catalytically inactive Cas variants allows modulation of pathways involved in acidification, proteolysis, EPS production, and central metabolism, providing a level of control that surpasses traditional regulatory systems.

Before the widespread adoption of CRISPR-based approaches, inducible expression systems played a foundational role in LAB biotechnology. The nisin-controlled gene expression (NICE) system became the most extensively used platform due to its strong inducibility and reliance on a food-grade signal molecule [72,76,109,110]. Additional regulatory systems based on substrates such as lactose or xylose, as well as environmental triggers, including temperature or pH, further expanded the range of available toolkits for controlled expression in dairy applications [73,74,111,112]. These early platforms demonstrated the feasibility of using safe inducers for tightly regulated protein production and established the conceptual framework for the development of modern synthetic promoters and CRISPR-based regulatory architectures [113].

## 4. Applications of GM-LAB in the Food Chain and Human Health

Engineered probiotics have emerged as programmable microbial systems capable of sensing, responding to, and actively modulating the host environment [114,115,116]. Genome-edited LAB have been developed with improved metabolic performance, tailored production of bioactive compounds, and enhanced probiotic functionality [24,26,27,29,30,31,53,64,66,75,76,90,113,117,118,119,120,121,122,123,124,125]. GM-LAB have been studied to produce in situ cytokines, anti-inflammatory factors, and vitamins [55,67,74,120,126,127], supporting targeted molecule delivery and modulation of local immune responses [73,121,128,129,130]. LAB expressing heterologous enzymes or neuroactive metabolites, including γ-aminobutyric acid (GABA), have shown the potential to improve intestinal physiology and contribute to host well-being [75,131,132,133]. LAB engineered with antimicrobial peptides, antiviral factors, immunomodulatory proteins, or diagnostic biosensors illustrate the breadth of potential applications [26]. This overview demonstrates how the rational engineering of probiotics can enhance their functional properties beyond their native probiotic activity.

### 4.1. Improved Fermentation Performance

In LAB physiology, several metabolic pathways influence flavor formation and redox balance during fermentation, particularly those involving citrate and pyruvate metabolism. Citrate catabolism can generate key aroma compounds such as diacetyl, while pyruvate represents a central metabolic node connecting carbohydrate fermentation to the synthesis of organic acids, alcohols, and flavor molecules. Enzymes such as α-acetolactate synthase, acetolactate decarboxylase, and aldehyde/alcohol dehydrogenases regulate the distribution of pyruvate flux towards different end products. Within this network, enzymes such as *AldB* and *AdhE* play a crucial role in maintaining intracellular redox balance, tuning the conversion of aldehyde and alcohol intermediates.

Targeted inactivation of *AldB* in *Lactococcus lactis* has been used to redirect metabolic flux towards α-acetolactate accumulation and increased diacetyl formation, thereby enhancing the buttery aroma of fermented dairy products [134,135]. Additional strategies include the deletion of *pip* genes to increase phage resistance and the removal of genes involved in biogenic amine production to reduce their accumulation [122,136,137,138,139,140]. In *Lactobacillus (Lb.) delbrueckii* subsp. *bulgaricus*, the generation of lactose-negative mutants has been exploited to fine-tune yogurt acidification profiles [141]. The introduction of citrate-positive phenotypes has also expanded metabolic capacities relevant to flavor development in cheese-making systems [142]. Proteolysis engineering, through transfer or overexpression of peptidases such as PepN, PepC, PepX, and PepI, has improved amino acid release during cheese ripening [143], while controlled lysis systems have further enhanced flavor development [144].

Within the context of technological and rheological features, EPS production has also been enhanced through the transfer of complete EPS gene clusters, improving the viscosity and texture of fermented dairy products [145]. Additionally, the removal of residual antibiotic resistance markers has improved the regulatory acceptance of modified starter cultures [26,50,146,147].

In addition to traditional dairy fermentations, and in line with current sustainability goals, engineered LAB are being explored for the valorization of lignocellulosic biomass and plant-derived substrates, expanding their role within emerging biorefinery and circular bioeconomy frameworks. A metabolically engineered *Lactiplantibacillus (Lp.) plantarum* strain was shown to efficiently convert lignocellulosic biomass into optically pure (>99.5%) D-lactic acid [148]. D-lactate is a key monomer for stereo-complex polylactic acid (PLA), a biodegradable polymer with enhanced thermal and mechanical properties. The authors achieved knockout of the *ldhL* gene (encoding L-lactate dehydrogenase) and introduced the xylose assimilation plasmid *pCU-PxylAB*, thereby enabling efficient utilization of the pentose sugars present in pretreated corn stover and sorghum stalks [148]. Nevertheless, despite these encouraging results, the authors noted that the reliance on antibiotic resistance markers remains a bottleneck for industrial-scale deployment [148].

### 4.2. Nutritional Enhancement of Foods and Bioactive Metabolite Synthesis

The introduction of heterologous genes has enabled LAB to acquire novel technological functions. Examples include phage-resistance operons, EPS biosynthesis clusters transferred between *Streptococcus (S.) thermophilus* and *L. lactis* [149,150], and metabolic engineering to enhance vitamin production [151,152,153], as well as lactose or citrate utilization. LAB have also been engineered to produce industrially relevant enzymes such as amylases, phytases, and xylitol-producing dehydrogenases, broadening their applications in both dairy and plant-based fermentations. Engineering of LAB for modulation of LA optical purity has been achieved through the deletion of *ldhD* or replacement with *ldhL*, yielding marker-free strains producing highly pure L-lactate suitable for PLA synthesis [148]. GM-LAB can be exploited as in situ producers of vitamins and bioactive compounds that enhance the nutritional profile of fermented foods. Engineering of folate, riboflavin, and cobalamin pathways has yielded strains capable of significantly increasing the vitamin content in dairy and cereal-based products [152,154,155]. Other engineered LAB produce GABA, bioactive peptides, or EPSs with potential health benefits, enabling the development of functional foods with added value [145].

### 4.3. Food Safety Improvement and Biopreservation

Recent studies have expanded the metabolic engineering of LAB by optimizing pathways for 3-hydroxypropionic acid, lactate-derived biochemicals, and other platform chemicals [39]. These works demonstrated how the regulation of redox balance, overexpression of dehydrogenases, and elimination of competing pathways can significantly increase yields of industrially relevant compounds. Moreover, these findings reinforce the feasibility of LAB as sustainable microbial chassis for biorefinery applications.

Biopreservation is another major application area for GM-LAB. Overexpression of bacteriocins such as nisin [156], pediocin, or bactofencin A [157] can enhance the inhibition of spoilage organisms and foodborne pathogens, thereby extending shelf life and improving safety [158]. LAB have also been engineered to degrade or bind undesirable compounds, including biogenic amines, mycotoxins, and environmental contaminants [158,159,160], thus reducing consumer exposure to these hazards.

### 4.4. Application of Engineered Probiotics In Vivo

Through improved functional capabilities such as adhesion, stress tolerance, and immunomodulatory activity, GM-LAB have demonstrated how the manipulation of surface proteins, sortase-dependent anchors, EPSs, and stress-response regulators can significantly enhance their persistence and interaction with host tissues. These genome-level interventions led to the development of GM-LAB strains with improved bioavailability and increased efficacy in mucosal delivery applications [73,121,128,129,130].

GM-*L. lactis* has emerged as a pioneering platform for mucosal delivery of cytokines and therapeutic proteins. Early studies demonstrated its capacity to secrete biologically active ILs while preserving correct folding and disulfide bond formation despite the absence of eukaryotic post-translational machinery [127,161,162,163]. Furthermore, in situ delivery has been shown to substantially reduce the required dose of effectiveness [162]. Comparable approaches have also been applied to antioxidant enzymes, including superoxide dismutase and catalase, further supporting the potential of GM-LAB in mitigating intestinal oxidative stress and inflammation [164,165,166].

In addition to constitutive delivery platforms, pathogen-responsive GM-*L. lactis* has been engineered through the incorporation of pheromone-sensing regulatory circuits, such as the pCF10-derived *prgX*/*prgQ* switch. This module enables sensing of the *Enterococcus (E.) faecalis* sex pheromone cCF10 and activation of a targeted antimicrobial response through the secretion of class IIa bacteriocins, resulting in effective inhibition of *E. faecalis* and *E. faecium*, including vancomycin-resistant isolates [117].

GM-LAB have also been evaluated as mucosal vaccine vehicles [167,168]. Vaccine efficacy is strongly influenced by antigen localization strategies, including secretion, cell wall anchoring, and cytoplasmic retention [169,170,171]. Surface display systems based on LPXTG sortase-dependent motifs [172], LysM domains [173], or S-layer proteins [174] enable stable presentation of viral, bacterial, and parasitic epitopes directly at mucosal interfaces. Surface-anchored antigens expressed in *L. lactis* and *Lactobacillus* spp. have consistently elicited robust mucosal IgA and systemic IgG responses in preclinical models [129,175,176,177,178,179]. Complementary approaches include strains expressing tetanus toxin fragments, HPV E7, M-protein epitopes, and antigens targeting pathogens such as *Clostridioides difficile* and *Helicobacter pylori*, supporting their feasibility as oral or intranasal vaccines [168]. In parallel, passive immunization strategies employing scFv-producing lactobacilli or *Streptococcus* species have demonstrated protective effects against *S. mutans* and *Candida albicans*, further expanding the immunotherapeutic scope of LAB-based systems [180,181,182].

### 4.5. GM-LAB as Cell Factories

Beyond their direct use in fermented foods, GM-LAB are increasingly used as microbial cell factories in closed bioreactor systems aimed at producing enzymes, flavors, sweeteners, antimicrobial peptides, and commodity chemicals such as lactic acid or 2,3-butanediol [15,183]. In these processes, viable GM cells and recombinant DNA can be removed during downstream processing so that, in some jurisdictions, the final purified ingredient may be placed on the market without GMO labeling [184]. This approach allows the benefits of genome engineering to be realized while minimizing regulatory and consumer acceptance challenges associated with viable GM-LAB in foods.

## 5. Benefits of GM-LAB Application

The application of GM-LAB in the agri-food chain offers a range of potential benefits that extend beyond the incremental technological improvements, encompassing process reliability, nutritional enhancement, sustainability, and safety. These benefits arise not from genetic modification per se, but from the ability to introduce targeted, predictable, and stable traits into microbial strains that are already well adapted to food and GI environments. When evaluated in a risk–benefit framework, GM-LAB represent a platform through which functional gains can be achieved with limited additional biological complexity compared to conventional starter cultures.

### 5.1. Technological and Economic Benefits

From an industrial perspective, one of the main advantages of GM-LAB is the improved level of process control. Engineered strains can exhibit more predictable acidification kinetics, greater tolerance to technological stresses (Table 1), reduced batch-to-batch variability, and improved fermentation performance (Table 2). Overall, these characteristics improve the reproducibility of fermentation processes, reducing the likelihood of batch failure and limiting the need for corrective interventions during production [184].

Increased microbial culture robustness can also be translated into measurable economic benefits, such as shorter fermentation times, reduced energy consumption, and more efficient use of raw materials. In large-scale food production, even relatively modest improvements in process stability can lead to significant cost savings. Furthermore, the ability to tailor strains to specific substrates or process conditions facilitates product diversification without requiring substantial modifications to existing production infrastructure.

### 5.2. Nutritional and Functional Benefits

GM-LAB enable the in situ production or the enhancement of nutritionally relevant compounds directly within fermented foods (Table 3). The products include vitamins, bioactive peptides, GABA, and EPSs, which may improve the nutritional profile or functional properties of foods [195]. Unlike post-fermentation fortification, microbial production during fermentation can improve bioavailability and reduce the need for synthetic additives.

In addition, targeted genetic modifications can reduce or eliminate undesirable components. These modifications support the development of fermented foods with tailored nutritional characteristics while maintaining high sensory quality.

### 5.3. Sustainability and Environmental Benefits

The use of GM-LAB has shown the potential to contribute to more sustainable food systems by increasing fermentation efficiency and extending product shelf life. Improved process efficiency can reduce waste generation along the food production chain and decrease the use of chemical preservatives and intensive thermal treatments, enhancing biopreservation in line with actual sustainability objectives and clean-label trends [24,204].

Moreover, engineered LAB capable of degrading or binding to contaminants can support the safer use of raw materials, potentially reducing food losses and improving overall resource utilization [205,206]. When applied in controlled environments, these traits can be integrated without increasing environmental exposure.

### 5.4. Safety Benefits

From a food safety perspective, GM-LAB can be designed to enhance intrinsic antimicrobial activity through increased or regulated production of bacteriocins and other inhibitory metabolites (Table 4). These traits may reduce the prevalence of foodborne pathogens and spoilage organisms, contributing to safer products and an extended shelf life.

In therapeutic and probiotic contexts, engineered LAB have demonstrated the capacity to deliver in situ bioactive molecules on mucosal surfaces, allowing the lowering of effective doses and, therefore, limiting systemic exposure. Importantly, many of the most advanced GM-LAB platforms incorporate biological containment strategies, such as auxotrophy or chromosomal integration of marker-free constructs, which further reduce potential risks associated with environmental persistence or HGT.

### 5.5. Evidence from Toxicological Studies

Although the overall number of toxicological studies on GM-LAB remains limited, available data from in vivo studies do not indicate an increased risk when strains are constructed using food-grade, marker-free systems and are thoroughly characterized. Multigenerational animal studies and controlled preclinical investigations have reported no adverse effects on growth, reproduction, organ function, or gut barrier integrity following oral administration of selected GM-LAB strains [213,214,215].

Taken together, these observations show that genetic modifications, when implemented in well-characterized LAB backgrounds and coupled with appropriate containment measures, do not intrinsically raise safety concerns. However, the relative scarcity of long-term studies and controlled human intervention trials highlights the need for continued and systematic evaluation before a wider adoption can be fully supported, particularly in applications involving the food chain. Indeed, the same genetic modifications that enable enhanced functionality may also be a threat to genetic stability, unintended metabolic effects, and interactions with the host or environment.

Within the EU, these aspects are addressed through a precautionary and highly structured regulatory framework, which requires a rigorous evaluation of potential risks before market authorization. As a result, the development and application of GM-LAB must be assessed within an integrated risk–benefit framework.

## 6. Risks, Safety Considerations, and EFSA Assessment

**Figure 3:**
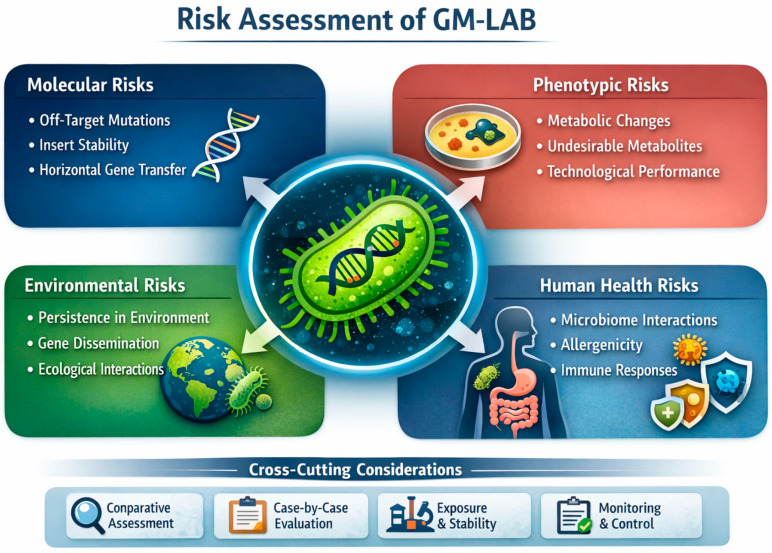
Conceptual framework for the safety assessment of GM-LAB. Risk evaluation in the EU considers molecular and genetic stability, phenotypic and metabolic effects, environmental impact, and potential implications for human health, including microbiome interactions and allergenicity.

From a biosafety and regulatory perspective, and according to the European Food Safety Authority (EFSA) classification scheme for genetically modified microorganisms intended for food and feed use [216], recombinant LAB engineered for surface display are categorized as Category 4 organisms (viable GMMs containing recombinant genetic material). Consequently, due to their involvement in multiple risks (Figure 3), they are subject to the most stringent environmental and safety assessments, including mandatory post-market environmental monitoring.

By contrast, wild-type LAB carrying non-covalently associated recombinant proteins may fall under Category 2, provided that the absence of recombinant DNA and viable genetically modified cells can be unequivocally demonstrated. In this case, the required environmental risk assessment is considerably less extensive and focuses primarily on confirming the absence of genetically modified material.

It is important to note that the EU labeling threshold of 0.9% GMO content applies only to the adventitious or technically unavoidable presence of authorized GM material. In EU legislation, this threshold is assessed per ingredient and is typically determined by quantitative PCR (qPCR) as the proportion of GMO-specific DNA relative to the total DNA of the corresponding ingredient. When GM-derived components are intentionally added, labeling requirements may apply regardless of the concentration. Consequently, the regulatory status of systems based on surface-displayed recombinant proteins must be evaluated on a case-by-case basis, depending on whether the final product contains or is considered to be produced from GM-derived material [217].

Under the EMA framework, recombinant bacterial strains are typically classified as gene therapy medicinal products, whereas non-GMO LAB with externally produced recombinant proteins may instead be regulated as biological medicinal products, bypassing GMO-related environmental requirements [218].

Provided that no recombinant DNA is involved, EU Directive 2001/18/EC declares that organisms obtained through mutagenesis—including chemical, UV, or antibiotic selection methods—are exempt from GMO legislation [33].

Pheromone-induced antimicrobial systems offer notable biosafety advantages because their activation occurs only in the presence of the target pathogen, reducing non-specific selective pressure on commensal microbes [117]. This controlled activation aligns with EU requirements for environmental containment and supports regulatory acceptance of GM-LAB engineered for pathogen-specific interventions. LAB-based vaccines exhibit strong safety profiles due to their non-pathogenic nature and extensive history of safe consumption. GM-LAB used as mucosal vaccine vectors do not persist in the GI tract and present minimal risk of HGT when engineered without antibiotic markers [91,219,220], supporting regulatory acceptance in food-grade contexts.

Safety evaluations indicate that GM-LAB designed with containment strategies and marker-free systems exhibit favorable safety profiles, with no adverse effects observed in preclinical or clinical studies [75,221,222]. Despite strong evidence of safety, public acceptance of GM probiotics remains a major challenge. Marker-free genome-editing approaches are considered particularly suitable for food-related applications, as they avoid the persistence of antibiotic resistance genes and reduce regulatory concerns [24].

### 6.1. Molecular and Genetic Risks

A major advance in GM-LAB biosafety was achieved through construction of the auxotrophic *L. lactis* Thy12 strain in which the *thyA* gene, encoding thymidylate synthase, is replaced with a therapeutic gene such as human IL-10 [221]. This deletion renders the strain strictly dependent on exogenous thymidine and leads to irreversible thymidine-less death under environmental conditions, providing an intrinsic biological containment mechanism [223]. This marker-free, plasmid-free system represented a significant regulatory milestone, supporting the approval of the first clinical trial, around 2006, using a live GM microorganism as a therapeutic agent [127]. Ten patients underwent treatment with capsules containing about 10^10^ CFU of lyophilized LL-Thy1212 twice daily for 7 consecutive days. Among these, one patient withdrew from the study because of persistent vomiting, whereas eight patients reported an improvement in clinical measures, with five reporting clinical remission. The cohort was followed for an additional 3 weeks (up to day 28) without any further adverse effects [127].

In mice, a 6-week oral administration study using *L. lactis* NZ9000/pNZPNK demonstrated the absence of toxicological effects, as no mortality, clinical symptoms, or behavioral changes were observed [224]. Body and organ weights remained comparable to those of controls, and hematological and biochemical parameters stayed within physiological ranges. Importantly, no bacterial translocation to blood or internal organs was detected, indicating preserved intestinal barrier integrity [224]. These findings confirm that this GM strain meets key EFSA toxicological criteria, including non-toxicity, absence of infectivity, and lack of systemic dissemination.

CRISPR-based editing introduces new considerations for risk assessment. Although off-target events occur at low frequency, they must be evaluated to ensure the absence of unintended genomic alterations [225]. Marker-free edited strains produced through RNP-mediated delivery or self-targeting plasmids can achieve high genetic stability, but verification of the absence of residual CRISPR elements is necessary to confirm safety [225,226]. While CRISPR generally reduces reliance on plasmids, some delivery systems still require transient plasmid vectors, necessitating assessment of potential HGT under fermentation or GI conditions. RNP-based strategies largely eliminate this concern and may be compatible with future regulatory differentiation between edited and transgenic organisms [227,228].

Safety evaluation of GM-LAB must carefully consider the origin and characteristics of the genetic elements introduced [229,230]. Lindgren highlighted that vectors and marker genes must be derived from the host species or GRAS organisms and that antibiotic resistance markers are not acceptable for food-grade applications [49]. Chromosomal integration through double-crossover events is preferred due to its stability and limited potential for HGT [231,232]. The genetic construct must not interfere with essential technological host traits or introduce biologically active products that could alter gut microbial ecology [113]. Renault’s analysis further emphasized several molecular safety concerns relevant to GM-LAB [122].

At the molecular level, key risks associated with GM-LAB include unintended insertions or deletions, off-target effects of genome editing, and the presence of mobile genetic elements or antibiotic resistance markers that could be horizontally transferred to other microorganisms [233]. The use of marker-free systems, chromosomal integration, and whole-genome sequencing helps in mitigating these risks.

### 6.2. Phenotypic and Metabolic Risks

Updated regulatory assessments highlight that GM-LAB and bifidobacterial strains engineered for therapeutic delivery must comply with strict EU GMO rules, even when modifications are marker-free or mimic natural recombination [118].

Current evidence supports their safety, given the lack of persistence, absence of toxin genes, and low HGT potential. Nevertheless, EU frameworks require detailed molecular characterization, containment strategies, and food-grade vector design before approval.

Phenotypic and metabolic risks arise from potential changes in substrate utilization, metabolite profiles, or stress responses, which may lead to the accumulation of undesirable compounds, altered organoleptic properties, or reduced strain robustness. Comprehensive characterization of growth, fermentation performance, and metabolite production is necessary to identify and manage such unintended effects (Table 5).

### 6.3. Environmental Risks

Because of their different localizations, antigens exhibit markedly different levels of exposure, stability, and environmental interactions. Antigen localization influences biosafety considerations in GM-LAB. Surface-anchored antigens increase immunogenicity but may pose higher ecological or microbiome interaction risks. Cytoplasmic localization, by contrast, restricts antigen exposure until bacterial lysis, reducing environmental interaction and potentially lowering biosafety concerns [71]. Precision editing technologies such as ssDNA recombineering introduce unique considerations for molecular risk assessment. Although the absence of foreign DNA and antibiotic resistance markers improves safety profiles, the possibility of unintended base substitutions or mismatch repair evasion requires careful validation. Additionally, the reliance on endogenous or heterologous recombinases necessitates verification that no residual recombination functions persist after strain modification [64].

While minimizing off-target survival, CRISPR-assisted editing may introduce double-strand breaks that require monitoring for unintended genomic rearrangements. Overall, precision genome editing aligns well with safety-by-design principles but must be accompanied by a comprehensive genomic characterization [113].

Therapeutic applications of GM-LAB have driven the development of robust biosafety strategies validated in animal models and clinical settings, confirming non-persistence after treatment cessation [91,95]. Containment approaches also include the removal of resistance markers and stable chromosomal integration, reducing HGT risk and aligning with EU regulatory requirements [49,217,218].

Environmental risk assessment of GM-LAB includes evaluation of (i) colonization potential, (ii) gene stability during fermentation, and (iii) likelihood of HGT in both food matrices and the GI tract. Experimental studies have demonstrated that, under typical fermentation conditions, plasmid-encoded traits may exhibit structural or segregational instability but do not transfer to other microorganisms [238,239]. This suggests a low risk of engineered gene dissemination.

Nevertheless, heterologous constructs require full assessment due to the uncertainty regarding their ecological impact and the theoretical possibility of transfer to related genera. Environmental risk assessment therefore focuses on survival, dissemination, and potential impact on microbial ecosystems once released via food production, effluents, or fecal excretion. This includes the consideration of natural transformation, bacteriophage-mediated gene transfer, and genomic plasticity within LAB populations and surrounding microbial communities [218].

### 6.4. Human Health Risks

Potential human health risks associated with GM-LAB must be evaluated within the broader context of the host–microbe interaction and within the ecological complexity of the human microbiome [240,241,242]. The introduction of targeted genetic modifications may alter microbial behavior, metabolic activity, or host interactions in ways that require a careful assessment before application in the food chain.

One of the primary concerns relates to the possible impact of GM-LAB on the structure and function of the human microbiome [243]. The gastrointestinal tract (GIT) hosts highly diverse microbial communities that play essential roles in digestion, immune regulation, and protection against pathogens [244]. Thus, perturbances in microbial composition—defined as dysbiosis [245]—have been associated with a variety of chronic conditions, including inflammatory bowel diseases (IBDs), metabolic disorders, and immune-mediated diseases [246,247,248,249,250]. The introduction of engineered microorganisms could theoretically influence these microbial ecosystems through competition, metabolic cross-feeding, or modulation of signaling pathways among microbial populations. Even subtle alterations in microbial metabolic outputs or ecological niches may influence host physiology, particularly in vulnerable populations such as infants, elderly individuals, or immunocompromised patients. Similar concerns have been raised in broader analyses of GMOs, where engineered microbes may interact with host microbiomes and influence microbial equilibrium or metabolic networks [251,252].

Another important consideration concerns the expression of heterologous proteins or bioactive compounds by engineered LAB. Genetic modifications designed to enhance probiotic functionality or to deliver therapeutic molecules may lead to the production of proteins that have not previously been present in food matrices [253]. Such molecules could potentially trigger immune responses, allergenicity, or unintended biological activity [254,255]. Although most GM-LAB developed for food or therapeutic purposes are designed by using food-grade and marker-free genetic systems, the biological properties of newly expressed proteins must still be evaluated through molecular characterization, toxicological testing, and digestibility studies [243]. In fact, proteins exhibiting structural similarity to known allergens or that interact with host immune pathways may require detailed immunological evaluation.

Metabolic alterations introduced through genetic engineering may also affect host exposure to microbial metabolites. LAB naturally produce a range of organic acids, bacteriocins, and signaling molecules that contribute to food fermentation and microbial competition [256,257,258]. However, targeted modifications may redirect metabolic fluxes or introduce new biosynthetic pathways, potentially resulting in the accumulation of metabolites with unknown physiological effects. Engineered microbial metabolism has been shown in other systems to influence host–microbe interactions through the production of signaling compounds, intermediates of central metabolism, or antimicrobial molecules that can affect microbial community dynamics [259]. For this reason, comprehensive metabolomic profiling and physiological characterization are often recommended as part of safety assessment strategies.

HGT represents another theoretical risk associated with viable GMO [242]. Although LAB are not typically considered to harbor highly mobile genetic elements compared with certain environmental bacteria, gene transfer events may occur under specific conditions within the GI environment. Such events could potentially involve the transfer of engineered DNA sequences to other members of the gut microbiota [260,261]. The likelihood of such transfer is generally reduced by using chromosomal integration, marker-free constructs and avoiding antibiotic resistance genes in food-grade systems. Nonetheless, regulatory frameworks require evaluation of the stability of inserted genetic elements and their potential mobility within microbial communities.

Finally, the persistence and colonization capacity of GM-LAB should be considered in risk assessment. Most probiotic LAB strains exhibit transient colonization, with their cell density gradually reducing after their intake. However, modifications that improve stress tolerance, adhesion, or metabolic capabilities could theoretically enhance survival or persistence within the host. While such traits may be advantageous for probiotic efficacy, they also require evaluation to ensure that engineered strains do not establish long-term ecological changes within the gut microbiome.

## 7. Regulatory Framework for GM-LAB in the Food Chain

In the EU, the regulatory assessment of GM-LAB is shaped by the interaction between established GMO legislation, the rapid evolution of genome-editing technologies, and societal perceptions of risk and benefit. Although LAB have a long history of safe use in food fermentation and many species are recognized as GRAS or included in the QPS list, the introduction of targeted genetic modifications places GM-LAB within one of the most stringent regulatory environments worldwide. The following paragraphs outline the current EU regulatory framework, the classification of engineered LAB products, and the broader international and societal context influencing their potential adoption.

### 7.1. EU Regulatory Framework for GM-LAB

The EU regulatory framework governing GM-LAB is primarily based on Directive 2001/18/EC for the deliberate release of genetically modified organisms into the environment and Regulation (EC) No. 1829/2003 for genetically modified food and feed. Under this legislation, any viable GM-LAB intended for use as starter cultures, probiotics, or live delivery systems must undergo a comprehensive, case-by-case risk assessment prior to authorization. This assessment, coordinated by the EFSA, covers molecular characterization and genetic stability, together with phenotypic properties, toxicological and allergenicity profiles, nutritional impact, and potential environmental effects.

A keystone concept in EU food safety assessment deals with substantial equivalence; in fact, GM-LAB strains may be considered substantially equivalent to their non-modified counterparts when no relevant differences affecting safety or nutritional value are identified. Substantial equivalence, however, does not represent a declaration of safety; rather, it determines the scope and depth of additional testing requirements. When equivalence cannot be established, the assessment focuses on the specific traits introduced by genetic modification, including the nature of the genetic construct, its long-term stability, and the biological activity of any expressed products.

A major regulatory turning point occurred in 2018, when the Court of Justice of the European Union ruled that organisms obtained through directed mutagenesis, including genome-editing techniques such as CRISPR–Cas, fall under the scope of GMO legislation [122]. Therefore, genome-edited LAB are regulated under the same legal framework as transgenic strains, irrespective of whether foreign DNA is present in the final organism. This interpretation contrasts with the regulatory status of strains obtained through classical mutagenesis approaches—such as chemical or UV mutagenesis, antibiotic selection strategies, and adaptive laboratory evolution—which remain exempt from GMO legislation provided that no recombinant DNA is introduced. As a result, precisely edited, marker-free LAB strains may be subject to stricter regulatory requirements than strains carrying multiple uncharacterized mutations generated through traditional methods, highlighting a persistent asymmetry in EU risk governance.

### 7.2. Regulatory Classification of Engineered LAB Products

Beyond the legal definition of a GMO, the regulatory assessment of LAB-based products depends critically on how engineered strains are used and on the presence of viable genetically modified cells or recombinant material in the final product. EFSA classification frameworks for genetically modified organisms differentiate between products that contain viable genetically modified cells carrying recombinant DNA and those in which neither genetically modified organisms nor recombinant DNA can be detected in the final product.

Viable GM-LAB expressing recombinant genes are assigned to the highest regulatory category and are therefore subjected to comprehensive environmental risk assessment and, in most cases, mandatory post-market environmental monitoring. In contrast, non-GMO LAB associated with externally produced recombinant proteins that are non-covalently displayed on the cell surface, or immobilized on non-living bacteria-like particles, may be classified under lower-risk categories, provided that the absence of recombinant DNA and viable genetically modified cells can be clearly demonstrated. Nevertheless, the deliberate use of recombinant material generally activates labeling obligations and must be assessed within the relevant food or medicinal product regulatory frameworks.

A further distinction is drawn between food chain and therapeutic applications. LAB developed as live biotherapeutic products or as mucosal vaccine delivery systems are typically regulated under medicinal product legislation, where they may be categorized as gene therapy medicinal products or biological medicinal products rather than as foods. Experience from clinical development programs—such as those involving *L. lactis* strains engineered to secrete interleukin-10 for IBD—demonstrates that even non-pathogenic and non-persistent GM-LAB require extensive documentation addressing genetic stability, biological containment, and environmental safety prior to authorization. These examples emphasize the value of chromosomal integration, marker-free genetic designs, and safety-by-design approaches in facilitating regulatory acceptance.

Finally, GM-LAB are increasingly employed as microbial production platforms in closed fermentation systems for the manufacture of enzymes, metabolites, or purified bioactive compounds. In these contexts, viable genetically modified cells and recombinant DNA can be effectively removed during downstream processing. Depending on the regulatory jurisdiction and the characteristics of the final product, such ingredients may, in certain cases, be marketed without GMO labeling, highlighting how regulatory classification is driven primarily by product attributes rather than by the genetic engineering process itself.

## 8. Consumer Acceptance, Future Perspectives, and Research Gaps

Beyond the EU, regulatory approaches to genetically engineered microorganisms differ substantially. In several non-European jurisdictions, including the United States, greater emphasis is placed on the characteristics of the final product rather than on the technique used to generate it. Consequently, genome-edited microorganisms or products derived from GM cell factories may therefore be assessed under existing food or additive regulations without being classified as GMOs, provided that recombinant DNA is absent from the final product. These differences have contributed to an uneven global innovation landscape, potentially shifting research and commercialization of engineered LAB towards regions with more flexible regulatory frameworks.

In parallel with formal regulations, consumer perception remains a decisive factor for the practical deployment of GM-LAB in the food chain. Surveys consistently indicate that European consumers remain cautious towards GMOs, even when potential benefits for sustainability, safety, or health are highlighted. Notably, non-GMO approaches such as classical mutagenesis or adaptive laboratory evolution—despite their lower precision—are often perceived as more acceptable than modern genome-editing technologies. In this context, transparent communication, trust in regulatory institutions, and clear risk–benefit framing are essential prerequisites for any future integration of GM-LAB into European food systems.

Future research on GM-LAB should focus on integrating safe-by-design principles, in which hazard identification, risk reduction, and regulatory considerations are embedded into the early stages of strain development. Advances in whole-genome sequencing, systems biology, and computational modeling can support prediction and monitoring of unintended effects. There is a need for more comprehensive toxicological and ecological studies, including human intervention trials for probiotic GM-LAB, to expand the evidence base for safety assessment. On the regulatory side, ongoing discussions within the EU on the treatment of new genomic techniques may lead to a differentiated framework for certain categories of genome-edited organisms. Clarifying the status of engineered LAB will be essential to unlock the potential benefits of these technologies while maintaining high safety standards and consumer confidence.

## 9. Conclusions

LAB are indispensable key players in the food chain and attractive chassis for biotechnological innovation. Their natural safety, long history of use, and ease of genetic manipulation make them suitable candidates for improving food quality, enhancing fermentation performance, producing functional metabolites, and delivering therapeutic molecules. Genetic modification and new breeding techniques (NBTs) offer substantial opportunities to improve fermentation performance, enhance the nutritional and functional qualities of foods, and support the development of safer and more sustainable food systems. Recent progress in gene-editing technologies—particularly CRISPR-based tools, marker-free chromosomal integration strategies, and food-grade vector systems—has considerably broadened the range of feasible modifications. These advances enable the targeted engineering of metabolic pathways, surface display platforms, antimicrobial mechanisms, and immunomodulatory functions.

At the same time, the application of GM-LAB within the European Union remains limited by strict GMO legislation and by a cautious public perception. In practice, only strains developed using food-grade, antibiotic-free, and chromosomally integrated genetic systems are likely to align with current EU regulatory expectations. Moving forward, a balanced and evidence-based evaluation of both potential risks and benefits will be essential to inform decision-making regarding the use of GM-LAB in the food chain. Aligning regulatory frameworks with scientific advances, strengthening the safety evidence base, and fostering transparent engagement with consumers will be critical factors in determining whether—and under what conditions—GM-LAB can be responsibly incorporated into European food production systems.

## Figures and Tables

**Table 1:** Degree of GM-LAB improved tolerance to technological stresses.

Species	Outcome and Technological Stressor	Ref.
*Lc. casei*	Improved biomass by 60% at a pH of ~4	[185]
*Lb. gasseri*	Improved cell density at 1.4 mM H_2_O_2_: +30%	[186]
*Lb. acidophilus*	Improved cell density at 1.4 mM H_2_O_2_: +90%	[186]
*Lc. rhamnosus*	Resistance (survival) to FTG: +40–90%	[187]

Abbreviations: *Lb.*, *Lactobacillus*; *Lc.*, *Lacticaseibacillus*; FTG, freeze–thaw–growth.

**Table 2:** Rates of improved fermentation performance achieved by GM-LAB.

Species	Improved Fermentation Performance	Ref.
*L. lactis*	L-lactate: ≈+150%	[188]
*Lb. helveticus*	L-lactate: ≈+20%	[189]
*Lb. thermophilus*	L-lactate: ≈+16%	[190]
*Lc. paracasei*	Total (D-/L-) lactate: ≈+3.7% L-lactate OP (95.4% ⟶ 99.1%)	[191]
*L. lactis*	Diacetyl: ≈+289%	[192]
*Ls. reuteri*	1, 3-propanediol: +34%	[193]
*S. thermophilus*	Acetaldehyde: ≈+80–90%	[194]

Abbreviations: L., Lactococcus; Lb., Lactobacillus; Lc., Lacticaseibacillus; Ls., Limosilactobacillus; S., Streptococcus; OP, optical purity.

**Table 3:** Nutritional improvements achieved by GM-LAB.

Species	In Situ Metabolite Production	Ref.
*Ls. reuteri*	folate	[196]
*L. lactis*	riboflavin	[197]
*Lp. plantarum*	riboflavin	[198]
*L. lactis*	xylitol	[199]
*L. lactis*	mannitol	[200]
*Lc. casei*	sorbitol	[201]
	**Degradation of Harmful Substances**	
*Ls. reuteri*	Zearalenone	[202]
*Lp. plantarum*	Tannins	[203]

Abbreviations: L., Lactococcus; Lc., Lacticaseibacillus; Lp., Lactiplantibacillus; Ls., Limosilactobacillus.

**Table 4:** Degradation of harmful substances carried out by GM-LAB.

Species	In Situ Antimicrobial Molecule Synthesis	Ref.
*L. lactis*	Lacticin 3147	[207]
*L. lactis*	Nisin, lacticin 3147	[208]
*Lp. plantarum*	Plantaricin	[208]
*L. lactis*	Nisin	[209,210,211]
*L. lactis*	cLFchimera	[212]

Abbreviations: L., Lactococcus; Lp., Lactiplantibacillus.

**Table 5:** Overview of data requirements for EFSA risk assessment of GM-LAB.

Assessment Dimension	Key Data Requirements	Relevance for GM-LAB	Typical Evaluation Approach	EFSA Reference
Molecular characterization	Insert sequence and structure, copy number, genetic stability, absence of vector backbone and unintended sequences	Confirms genetic integrity and absence of unintended modifications	Whole-genome sequencing (WGS), PCR, Southern blot, bioinformatics analyses	EFSA, 2011 [216]; EFSA, 2021 [234]
Phenotypic characterization	Growth kinetics, metabolic activity, stress tolerance, technological performance	Identifies unintended phenotypic changes affecting functionality or safety	Fermentation assays, metabolic profiling, stress response tests	EFSA GMO Panel, 2011 [216]; EFSA, 2023 [234]
Toxicological assessment	Toxicity, pathogenicity, allergenicity potential	Ensures safety for human and animal consumption	In vitro assays, in vivo studies, bioinformatic allergenicity assessment	EFSA, 2011 [216]; EFSA, 2017 [235]
Nutritional assessment	Composition, bioavailability, nutritional equivalence	Verifies equivalence to non-GM comparator	Comparative compositional analysis, nutrient bioavailability studies	EFSA, 2011 [216]
Environmental risk assessment	Persistence, dissemination, ecological interactions, gene transfer potential	Evaluates environmental impact and ecological safety	Survival studies, environmental exposure modeling	EFSA GMO Panel, 2011 [216]; EFSA, 2010 [236]
Horizontal gene transfer (HGT)	Mobility of genetic elements, transfer frequency, DNA stability	Assesses risk of dissemination of engineered DNA	Genomic stability analysis, in situ/in vitro HGT models	EFSA, 2011 [216]; EFSA, 2012 [237]
Post-market monitoring	Environmental and health surveillance after commercialization	Ensures long-term safety when required	Monitoring plans, surveillance systems, reporting frameworks	EFSA GMO Panel, 2011 [216]; EFSA, 2011 [216]

Abbreviations: GM-LAB, genetically modified lactic acid bacteria; HGT, horizontal gene transfer.

## Data Availability

No new data were created or analyzed in this study. Data sharing is not applicable to this article.

## Abbreviations

The following abbreviations are used in this manuscript:

ALE	Adaptive Laboratory Evolution
BLPs	Bacteria-Like Particles
CFU	Colony-Forming Units
CRISPR	Clustered Regularly Interspaced Short Palindromic Repeat
CRISPRa	CRISPR activation
CRISPRi	CRISPR interference
DSS	Dextran Sodium Sulfate
ECJ	European Court of Justice
EFSA	European Food Safety Authority
EMA	European Medicines Agency
EPSs	Exopolysaccharides
EU	European Union
GABA	γ-Aminobutyric Acid
GI	Gastrointestinal
GIT	Gastrointestinal tract
GM	Genetically Modified
GM-LAB	Genetically Modified Lactic Acid Bacteria
GMMs	Genetically Modified Microorganisms
GMO	Genetically Modified Organism
GRAS	Generally Recognized as Safe
HGT	Horizontal Gene Transfer
HPV	Human *Papillomavirus*
IBD	Inflammatory Bowel Disease
IJMS	*International Journal of Molecular Sciences*
IL	Interleukin
LAB	Lactic Acid Bacteria
LA	Lactic Acid
NBTs	New Breeding Techniques
NICE	Nisin-Controlled Gene Expression
PLA	Polylactic Acid
QPS	Qualified Presumption of Safety
RNP	Ribonucleoprotein
SCFAs	Short-Chain Fatty Acids
SICE	Stress-Inducible Controlled Expression
ssDNA	Single-Stranded DNA
SSCF	Simultaneous Saccharification and Co-Fermentation
TNBS	Trinitrobenzene Sulfonic Acid
XIES	Xylose-Inducible Expression System
